# The Effect of Arginine on the Phase Stability of Aqueous Hen Egg-White Lysozyme Solutions

**DOI:** 10.3390/ijms24021197

**Published:** 2023-01-07

**Authors:** Sandi Brudar, Barbara Hribar-Lee

**Affiliations:** Faculty of Chemistry and Chemical Technology, University of Ljubljana, Večna pot 113, SI-1000 Ljubljana, Slovenia

**Keywords:** hen egg-white lysozyme, arginine, cloud-point temperature, molecular dynamics, self-association, phase stability of protein solutions, force field effect

## Abstract

The effect of arginine on the phase stability of the hen egg-white lysozyme (HEWL) has been studied via molecular dynamics computer simulations, as well as experimentally via cloud-point temperature determination. The experiments show that the addition of arginine increases the stability of the HEWL solutions. The computer simulation results indicate that arginine molecules tend to self-associate. If arginine residues are located on the protein surface, the free arginine molecules stay in their vicinity and prevent the way protein molecules “connect” through them to form clusters. The results are not sensitive to a particular force field and suggest a possible microscopic mechanism of the stabilizing role of arginine as an excipient.

## 1. Introduction

The self-assembly of proteins is a common phenomenon occurring in almost all systems where protein molecules are present and is therefore receiving considerable attention in various disciplines dealing with protein solutions, such as chemistry, physics, medicine, pharmacy, material sciences, and food sciences [1]. Even though the protein self-assembly can in certain cases be beneficial, such as internal cell space organization [2], it could lead to the formation of protein aggregates that destabilize protein solutions and as such represent a major problem for the formulation of stable biological solutions [3]. Developing appropriate protein aggregation inhibition methods and excipients therefore represents an important field of research today [4,5,6].

One of the most commonly used excipients to suppress protein aggregation is L-arginine [7,8,9,10,11,12]. Even though the mechanism of the beneficial effect of arginine is still not completely understood, several experimental and theoretical studies have been conducted to elucidate its role. While in some cases arginine was found to act as a neutral crowder stabilizing proteins in their native form [7], other authors claim that no such evidence has been observed, and the presence of arginine only affects the kinetics of aggregation [8]. Another observation made by Shukla et al. based on their computer simulation studies [9] suggests that the self-association of arginine molecules plays an important role in its binding and inhibition of protein aggregation. The arginines in an aqueous solution, namely by themselves, tend to stack their methylene groups to form clusters with head-to-tail hydrogen bonding [13]. In the work of Tomita et al., however, the authors show that arginine residues bind to the protein surface and thereby reduce the stickiness of the protein molecules, making it less prone to aggregation [14].

In our previous work, the mechanism of self-association of several globular proteins was studied via a molecular dynamics (MD) simulation. By performing an extensive analysis of intermolecular residue–residue interactions, we showed that arginine is of paramount importance in the initial stage of the aggregation of the HEWL and γ-D crystallin [15]. The results suggest that the partial parallel and anti-parallel stacking of arginine methylene groups on different proteins occurs, stabilizing the initial protein contacts. From this, the hypothesis was formed that the free arginine molecules compete with the protein surface for arginine–arginine contacts, reducing their availability to form protein–protein complexes. In the present work, we tested this hypothesis further.

The paper is organized as follows. After this brief Introduction, the Results and Discussion are described, followed by the Materials and Methods section. The Conclusions are given at the end.

## 2. Results and Discussion

Even though it was suggested previously that a possible role of arginine as an excipient is to stabilize the native structure of the protein, thus preventing its unfolding [7], we limit ourselves in this work to investigate the role of free arginine molecules in preventing the aggregation of HEWL molecules in their native form.

### 2.1. Added Arginine Increments the Phase Stability of HEWL Solutions—Experimental Observation

**Figure 1:**
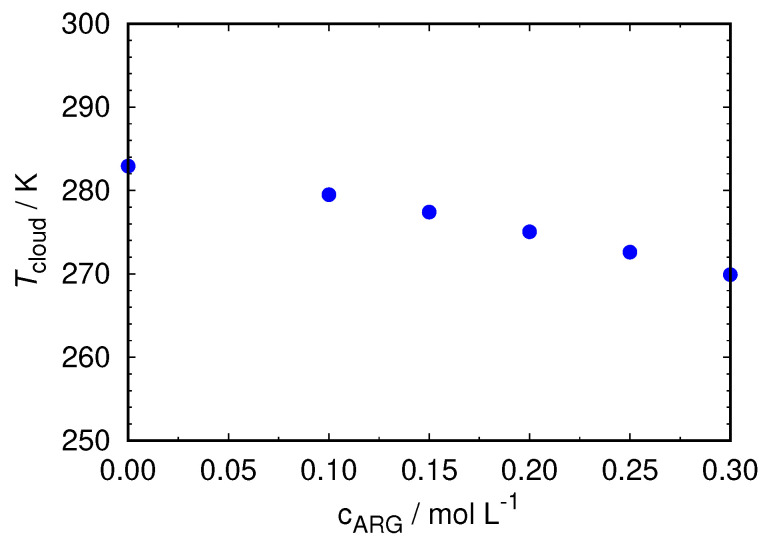
Measured $T_{\mathrm{cloud}}$ of 90 mg mL${}^{-1}$ HEWL in 0.1 M ACES containing 0.25 M NaBr at different concentrations of free arginine.

We begin by presenting the results for the experimentally measured phase stability of the HEWL solutions in the presence of free arginine molecules. It was established that the formation of protein aggregates in the solution leads to a liquid–liquid phase separation which can be detected by measuring the $T_{\mathrm{cloud}}$ of protein solutions [16,17]. Because it was shown before that different concentrations of arginine molecules can affect the stability of protein solutions through a different mechanism [8], we here determined how the $T_{\mathrm{cloud}}$ of HEWL solutions depends on the arginine concentration. The results are shown in Figure 1.

One can see that within the concentration range studied here, the $T_{\mathrm{cloud}}$ of the HEWL solutions decreases with an increasing concentration of free arginine molecules. A lower $T_{\mathrm{cloud}}$ means that the solutions would undergo a phase separation at lower temperatures and are therefore more stable. This is consistent with our hypothesis of free arginine competing to bind to arginine residues on the protein surface. The more free arginine molecules in the solution, the more efficiently they will conceal the hot spots on the protein, preventing them from interacting with other proteins forming aggregates. Next, we were interested in the microscopic mechanism of this phenomenon.

### 2.2. MD Simulations Show That Arginine Molecules Have a Strong Tendency toward Self-Association

**Figure 2:**
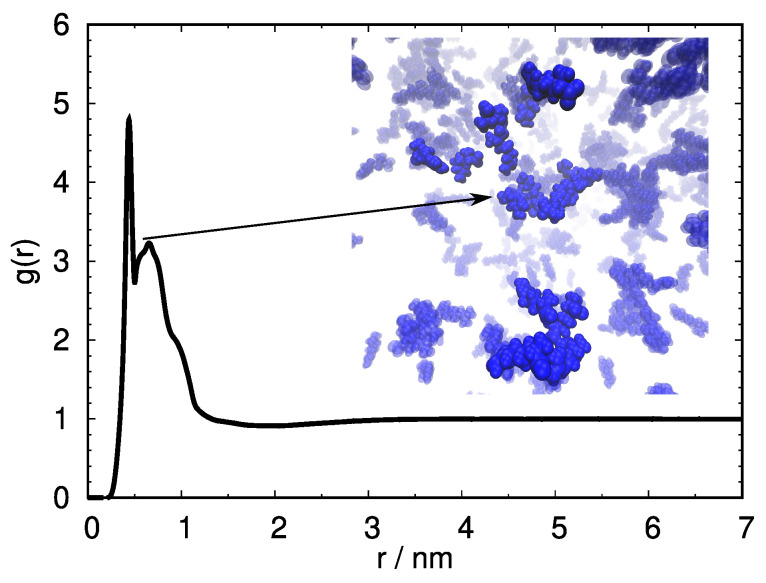
Arginine–arginine COM pair distribution function with a snapshot of arginine clustering from the simulated 0.3 M arginine solution at 267 K.

To further investigate the role of free arginine in determining the stability of HEWL solutions, we have next resorted to MD computer simulations. Because according to our hypothesis arginine–arginine interactions were crucial in the stabilization mechanism, we were first interested if this can be reflected in a computer simulation. We have therefore performed the simulation of pure arginine at the largest concentration that has been studied experimentally, namely 0.3 M. As expected, and suggested previously [9], the free arginine molecules show an extensive trend to self-associate, as seen from the arginine–arginine pair distribution function shown in Figure 2. Clusters of two and more arginine molecules are formed (see insert of Figure 2), connecting to each other mostly through head-to-tail hydrogen bonding and thus aligning their hydrophobic chains, the same mechanism that was previously observed in computer simulations of globular proteins [13,15].

#### 2.2.1. Addition of Arginine Reduces the Self-Association of HEWL

Encouraged by these results, we next performed the MD computer simulations using the same force field (i.e., OPLS; see the Materials and Methods for details) to study HEWL solutions in the presence and in the absence of free arginine molecules. The concentration of the added free arginine molecules was kept the same as in the case of the pure arginine simulation. The simulation conditions (*T* = 267 K and $\gamma_{\mathrm{HEWL}}$ = 93 mg mL^−1^) were chosen in the range where the HEWL solutions in the absence of arginine undergo phase separation [15].

**Figure 3:**
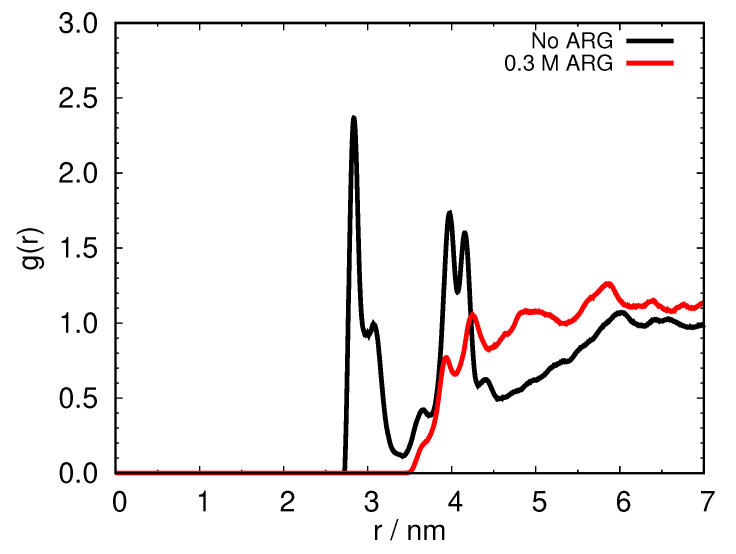
Protein–protein COM pair distribution function for 93 mg mL${}^{-1}$ aqueous HEWL solution at 267 K with (red) and without (black) added arginine.

As seen from Figure 3, the addition of arginine into the HEWL solution causes a distinct difference in the protein–protein pair distribution function. In the case where arginine is absent from the solution, the two protein molecules on average come much closer together (i.e., almost 1 nm) compared to the case where the 0.3 M free arginine is present in the solution. The radius of gyration for an HEWL molecule is approximately 1.38 nm [15]; this shows that in the case of no arginine, HEWL molecules come into close contact, while in the presence of free arginines no self-association of HEWL occurs.

**Figure 4:**
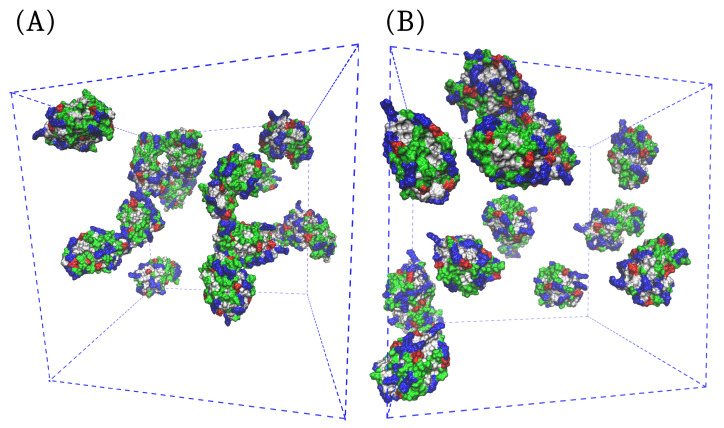
Snapshots from the computer simulations showing just HEWL molecules (**A**) without the presence of free arginine and (**B**) with added 0.3 M arginine.

**Figure 5:**
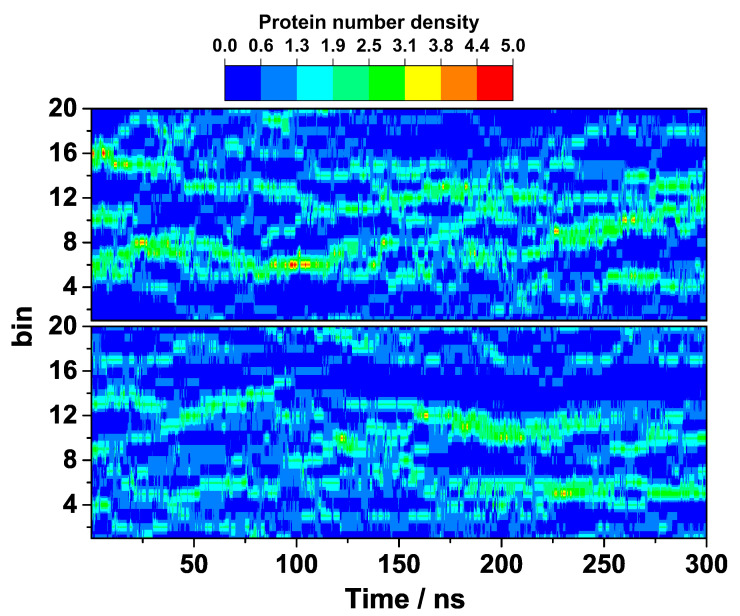
Density fluctuation of HEWL without (**top**) and with (**bottom**) added arginine in the x-axis direction.

The difference in the distribution of HEWL molecules within the two solutions can be even more clearly seen from the combination of the visual analysis and density fluctuations of both simulations (Figure 4 and Figure 5). The snapshots in Figure 4 demonstrate that in the absence of arginine (Figure 4A), large thread-like HEWL clusters consisting of up to five molecules are present at several times in the simulation, while in the presence of free arginine molecules, the HEWL molecules are more or less uniformly distributed as monomers and occasional dimers within the solution (Figure 4B). On the other hand, the self-association of free arginine molecules does not seem to be particularly influenced by the presence of HEWL molecules, as arginine molecules are numerous and have a large tendency to form clusters. Nevertheless, it was noticed that clusters consisting of free arginine tend to be slightly smaller in solutions containing HEWL molecules than in the pure arginine solution, which is due to the high binding preference of free arginine to residues on an HEWL surface. Also noteworthy is the finding that the conformation of HEWL does not change during self-assembly, which is consistent with the phenomenon of the liquid–liquid phase separation.

A better insight into the suppressed HEWL self-assembly by arginine during the course of the entire simulation is provided by the calculated local density fluctuations of both simulations. The local density of the HEWL molecules, ΔN(x,t), defined as the number of the centers of mass of the protein molecules found at time t in a slab of width Δx, was computed from the MD trajectories, where Δx was 0.8 nm. The obtained results are shown as a heat map in Figure 5. In the absence of arginine (Figure 5, top), one can observe that the population of the HEWL dimers begins to grow very rapidly from the very beginning. This dimerization seems to be only partially reversible as more and more viable dimers begin to form and even start colliding together in larger clusters of up to five HEWL molecules at around 100 ns. Later on, these large assemblies that are responsible for a very clear two-phase protein region partially disintegrate, but clusters the size of at least HEWL trimers persist in this solution until the end. In the presence of arginine, this is not the case as the solution appears to be more homogeneous. Even though small protein associates, mostly in the form of dimers and trimers, occasionally form in the solution, they are not stable and would dissociate again in the course of the simulation (Figure 5, bottom).

**Figure 6:**
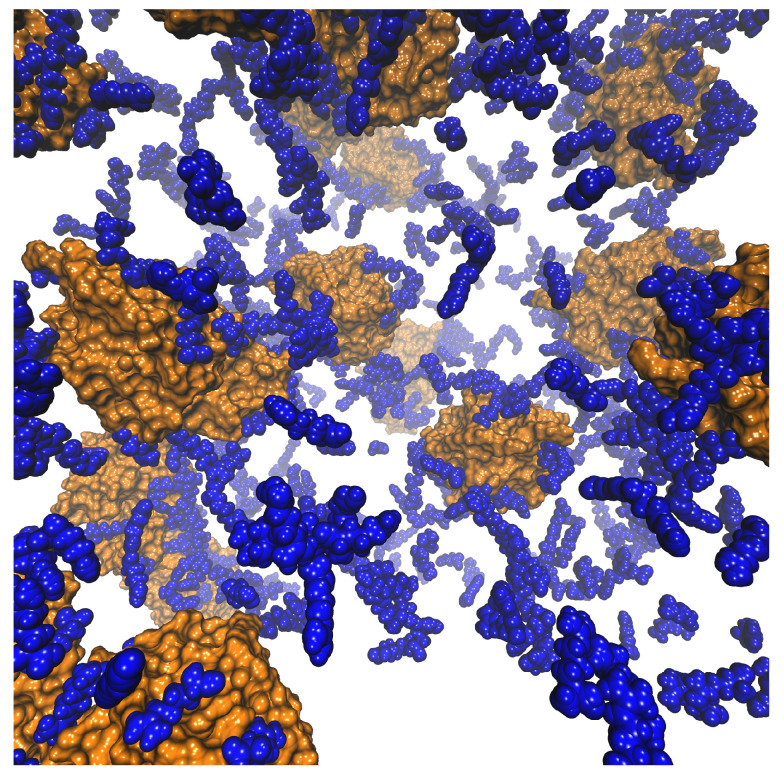
Snapshot of the 93 mg mL${}^{-1}$ aqueous HEWL solution at 267 K with added 0.3 M arginine. HEWL molecules are represented as orange surface, whereas arginine molecules as blue van der Waals spheres.

These results are all qualitatively consistent with the experimental observations, namely that free arginine molecules act as a stabilizing agent preventing protein aggregation. Note that a direct comparison between our simulations and experiments is not possible because no NaBr was added in the simulations. Within our calculations, we were not interested in the mechanism of arginine action in the presence of salt because the NaBr in our experiments was only used as a tool to reach the $T_{\mathrm{cloud}}$ conditions as in similar cloud-point studies [18,19]. To examine the mechanism of this stabilization further, we have analyzed the protein (HEWL molecule) surface–free arginine contacts in more details. A detailed snapshot of such interactions between the HEWL and arginine is shown in Figure 6. The protective role of arginine takes place in two paths. Firstly, in some cases, several larger clumps (even up to 10 arginine molecules) of arginine form in the solution and then attach to different parts of the protein surface, forming some kind of spacers that prevent it from sticking to other proteins. However, secondly, through a detailed visual analysis, one can notice that this is not the only way to protect against aggregation because monomeric arginines also attach to individual amino acids on the surface of proteins, which then, although already bound to proteins, attract other free arginines out of the solution and create a protective layer against other proteins. To discover which are the most important surface amino acids that act as the aforementioned targets for free arginine, we have calculated the fraction of the simulation time during which any arginine molecule is found in the vicinity of a particular amino acid residue on the surface of any protein molecule in the solution. The results are given in Table 1.

**Figure 7:**
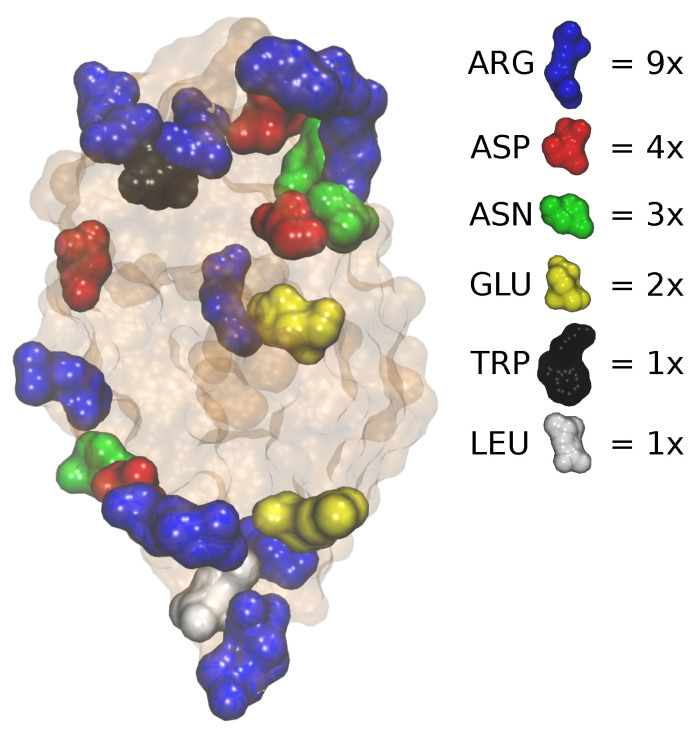
Visualization of the top 20 specific residues that were found to mostly interact with free arginine molecules.

One can see that free arginine molecules do not uniformly interact with the protein surface, but there are particular sites on the protein where they are more abundant, compared to other locations. These positions are visualized in Figure 7. Strikingly, these amino acids are situated at the protein surface locations that were previously identified to be involved in initiating the contacts between the HEWL molecules [15]. Another interesting observation that is also consistent with our previous results is the occurrence of a particular amino acid type among these locations; while an arginine residue was previously found to be particularly instrumental in facilitating the initial contacts between two protein molecules, it is also the most common residue found to be surrounded by arginine molecules from the solution (Figure 7 and Table 2).

#### 2.2.2. Changing the Force Field in Our Case Does Not Affect the Influence of Arginine on HEWL

One of the main concerns interpreting the MD simulation results is the sensitivity to the force field used. While several studies have been performed to validate various force fields against different experimental data (i.e., the structure and dynamics of folding, temperature-dependent structural propensities, etc.) [20,21,22,23,24,25], to the best of our knowledge, no such study has been carried out regarding the aggregation propensity of proteins. We therefore, before presenting reliable conclusions about the role of arginine, verified our results by repeating some of the simulations using a different force field, namely CHARMM27.

**Figure 8:**
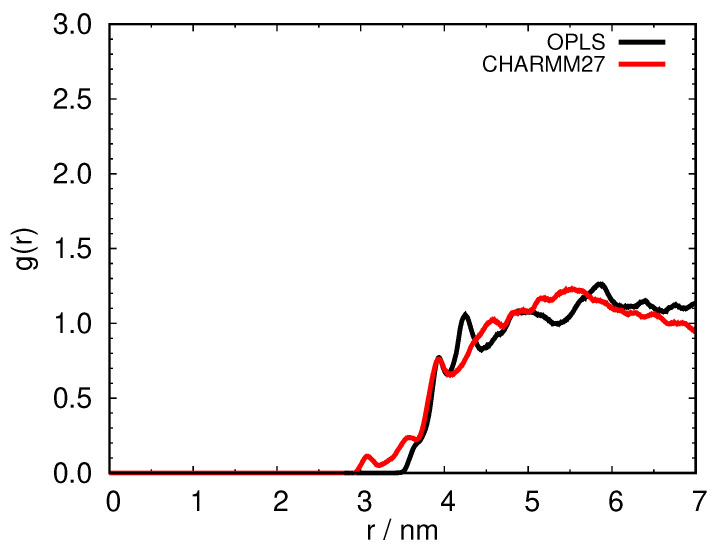
Protein–protein COM pair distribution function comparison between OPLS (black) and CHARMM27 (red) force field for 93 mg mL${}^{-1}$ aqueous HEWL solution at 267 K with added 0.3 M arginine.

First, we examined the protein–protein pair distribution function. Figure 8 shows the comparison between the two force fields used, and one can see that the two curves coincide within the statistical errors of the simulations. Regardless of the force field used, the presence of free arginine prevents the formation of protein–protein contacts.

**Figure 9:**
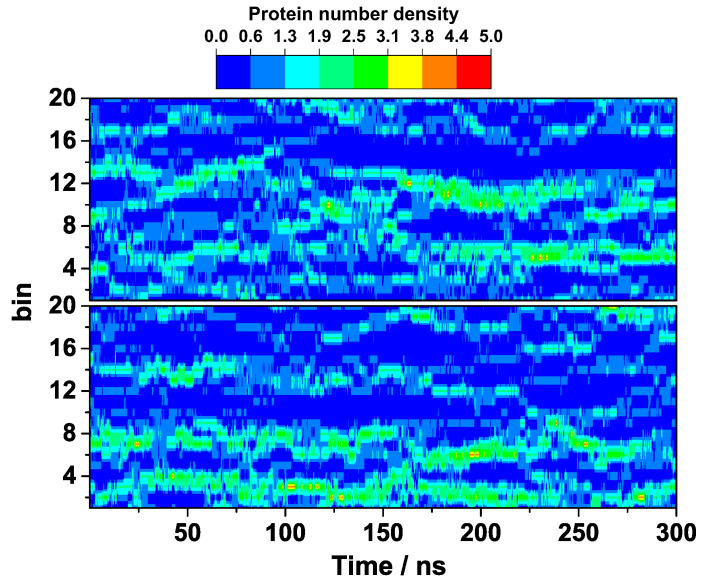
Density fluctuation of HEWL solution containing 0.3 M arginine with different force fields, namely OPLS (**top**) and CHARMM27 (**bottom**), in the x-axis direction.

The predicted stabilizing role of arginine was further checked by calculating the density fluctuations with an alternative force field (CHARMM27). From the results shown in Figure 9, it is clear that indeed no larger clusters of the HEWL molecules that would be particularly viable are formed in the presence of the 0.3 M arginine, regardless of the force field used.

## 3. Materials and Methods

### 3.1. Materials

Hen egg-white lysozyme (HEWL), sodium hydroxide, Spectra/Por^®^ float-a-lyzer^®^ G2 dialysis tubes, Amicon^®^ Ultra-15 centrifugal units, and sodium bromide were purchased from Merck (Darmstadt, Germany). L-arginine and 2-[(2-Amino-2-oxoethyl)amino]ethane-1-sulfonic acid (ACES) were obtained from Sigma-Aldrich (St. Louis, MO, USA).

### 3.2. Experimental Methods

#### 3.2.1. NaBr-ACES Arginine Solutions

To experimentally study the effects of arginine on the phase stability of HEWL solutions, we prepared 0.1 M ACES buffer at pH = 7.0 to prevent protein denaturation. Desired pH of ACES buffer was adjusted by carefully adding small amounts of 1 M sodium hydroxide. The final ACES solution was filtered through Sartorious filters with a pore size of 0.45 μm before further usage. NaBr was first thoroughly dried for 2.5 h in the presence of P_2_O_5_ at 107 °C. NaBr was then dissolved in ACES buffer to create several stock NaBr-ACES solutions that contain twice the concentration of NaBr (0.5 M) then later intended for cloud-point measurements. In all but one of these NaBr-ACES solutions, twice their later predicted concentration of arginine was then dissolved. All stock NaBr-ACES arginine solutions had their pH values checked and corrected with 1 M sodium hydroxide to obtain same values as for pure ACES buffer.

#### 3.2.2. HEWL-ACES Solutions

Phase separation of HEWL was investigated in the intermediate concentration regime (90 mg mL^−1^); therefore, a stock solution of 200 mg mL^−1^ of HEWL in 0.1 M ACES buffer was prepared. HEWL concentrations were determined optically by taking into account its extinction coefficient of 2.64 mL mg^−1^ cm^−1^ at 280 nm [26]. After HEWL was completely dissolved in ACES buffer, a thorough dialysis against pure ACES buffer followed at room temperature by using 5 mL Spectra/Por^®^ float-a-lyzer^®^ G2 dialysis tubes with a 3.5 kDa cut-off. The dialysis buffer was exchanged three times within 24 h. Due to certain amount of sample dilution after dialysis, HEWL-ACES solutions were repeatedly concentrated, using 15 mL Amicon^®^ Ultra-15 centrifugal units at 5000 rpm and 4 °C, until HEWL reached a concentration of 180 mg mL^−1^.

#### 3.2.3. Cloud-Point Measurements

The cloud-point temperature, $T_{\mathrm{cloud}}$, is a specific temperature at which the protein solution undergoes phase separation into two coexisting phases, either liquid–liquid or liquid–solid phases. Experimentally, $T_{\mathrm{cloud}}$ can be characterized as the temperature at which the first opacification of the protein solution occurs upon its cooling. In our study, such experiments were performed spectrophotometrically using Cary 100 Bio spectrophotometer (Varian, Australia). NaBr-ACES solutions with different additions of arginine and HEWL-ACES solutions were filtered through 0.2 and 0.45 μm filter pores (Sartorious), respectively. HEWL-ACES and NaBr-ACES arginine solutions were mixed together in a 1:1 ratio moments before the measurement and then transferred into black-walled quartz cuvettes with a pathlength of 1 cm and volume of 1 mL. The final concentrations of arginine in different cuvettes ranged between 0 and 0.3 M; meanwhile, HEWL, NaBr, and ACES final concentrations were identical in all cuvettes, namely 90 mg mL^−1^, 0.25 M, and 0.1 M, respectively. Afterward, solutions in each cuvette were subsequently cooled from 40 °C to around −5 °C, with a cooling rate of 0.1 °C min^−1^. In order to prevent condensation on cuvette walls, a constant flow of dry nitrogen was provided during cooling. Sample opacification accompanying phase transition was detected as an increase in measured absorbance at 340 nm.

### 3.3. MD Simulations

We performed atomistic MD computer simulations of aqueous solutions of a well-known globular protein, HEWL. Its crystal structure consists of 129 amino acid residues and was, in our case, taken from the Protein Data Bank (PDB) as 1aki.pdb [27]. The PDB structure of free arginine was created with Open Babel 3.0.0 [28] based on its canonical SMILES code, C(CC(C(=O)O)N)CN=C(N)N, obtained from PubChem [29]. The correct charge and structure of free arginine at pH = 7.0 was prepared interactively with the GROMACS simulation engine [30]. We decided to perform our MD study of HEWL at 93 mg mL^−1^ and 267 K in order to simulate this protein within its experimentally established liquid–liquid phase separation conditions [16,31]. To begin with our simulation protocol, we first minimized HEWL and free arginine structures with regards to their energy and then created three types of systems, all represented by a cubic simulation box of L = 16 nm. Namely, the first system consisted of only 93 mg mL^−1^ of HEWL (16 molecules), while in the second one, we kept the same protein content and added free arginine to reach its 0.3 M concentration (740 molecules). In the last system, the simulation box was filled with 740 free arginine molecules (0.3 M) without the presence of HEWL molecules. Then, all systems were solvated by using the SPC/E water model [32] and firstly equilibrated for 2 ns in the NVT ensemble and then also for 2 ns in the NPT ensemble. We used the OPLS force field [33] for all our calculations, and in the case of HEWL-arginine-containing solution, we also additionally tried the CHARMM27 force field to evaluate force field sensitivity [34]. Net charges of HEWL and arginine, namely +8 and +1, corresponding to pH = 7.0, were compensated by an adequate amount of Cl^−^ counterions to maintain electroneutrality of all solutions. Standard three-dimensional periodic boundary conditions were applied, and no buffer molecules or ions were included in simulated solutions as well. Production runs were performed in the NPT ensemble and the Parrinello–Rahman barostat maintained a pressure of 1 bar. All production runs lasted for 300 ns with a time step of 2 fs. Short-range electrostatic and van der Waals cut-off were both set at 1.0 nm; meanwhile, PME was used to treat long-range electrostatics.

## 4. Conclusions

In this paper, we have attempted to explain the experimentally observed role of molecular arginine to stabilize protein solutions by using MD computer simulations. While much effort has been made previously to clarify the part arginine plays in protecting native structures of proteins and preventing their unfolding, we here focused on its role as a suppressor of the self-association of proteins in their native structure. In the case of an HEWL, we have shown that molecular arginine tends to preferentially associate with other arginine molecules, as well as arginine residues on the HEWL surface. Its stabilizing role is thus twofold. Because arginine residues were previously found to frequently initiate contacts between protein molecules, a possible explanation of arginine’s role is that concealing these residues would slow down, or even suppress, the self-association process in protein solutions. At the same time, the camouflaging arginine molecules would further associate with other arginines in the solutions, forming some kind of spacers between the protein molecules, again preventing them from coming into contact. We plan to repeat similar calculations with other globular proteins to further test our conclusions.

## Figures and Tables

**Table 1:** List of specific amino acid residues near which (within 0.3 nm of distance) free arginines stay on average for the longest proportion of simulation time.

Res_ID_	Res_NAME_	Sim_TIME_/%
101	ASP	89.8 ± 2.3
18	ASP	77.8 ± 6.5
19	ASN	74.8 ± 5.9
128	ARG	71.5 ± 4.8
62	TRP	70.3 ± 5.9
73	ARG	68.5 ± 6.4
35	GLU	62.8 ± 7.3
21	ARG	57.7 ± 6.4
44	ASN	54.0 ± 4.6
61	ARG	53.5 ± 8.5
7	GLU	53.4 ± 6.9
14	ARG	53.1 ± 3.9
45	ARG	51.8 ± 6.4
125	ARG	51.7 ± 7.3
129	LEU	50.7 ± 5.7
68	ARG	50.2 ± 6.6
46	ASN	46.0 ± 8.6
48	ASP	43.9 ± 7.2
52	ASP	43.2 ± 9.7
112	ARG	42.9 ± 6.8

**Table 2:** List of top 10 amino acid types near which (within 0.3 nm of distance) free arginines stay on average for the longest proportion of simulation time. The column Num${}_{\mathrm{RESIDUES}}$ denotes the total number of a particular residue type in HEWL.

Res_NAME_	Total_TIME_/%	Num_RESIDUES_
GLU	58.1 ± 7.1	2
ARG	52.2 ± 6.6	11
ASP	46.4 ± 6.1	7
ASN	26.1 ± 4.5	14
PRO	23.9 ± 4.1	2
TRP	20.8 ± 3.5	6
GLN	20.3 ± 6.5	3
LYS	19.4 ± 4.1	6
GLY	16.8 ± 3.3	12
THR	15.6 ± 3.5	7

## Data Availability

Data are contained within the article.

## Abbreviations

The following abbreviations are used in this manuscript:

ACES	2-[(2-Amino-2-oxoethyl)amino]ethane-1-sulfonic acid
COM	center of mass
HEWL	hen egg-white lysozyme
MD	molecular dynamics
PDB	Protein Data Bank
PME	particle-mesh Ewald
$T_{\mathrm{cloud}}$	cloud-point temperature
